# Supplementary material for: Identification of photoperiod-induced specific miRNAs in the adrenal glands of Sunite sheep (Ovis aries)
Source: Front Vet Sci. 2022 Jul 22;9:888207. doi: 10.3389/fvets.2022.888207 (PMC9354845; doi:10.3389/fvets.2022.888207)
Supplement: Supplementary file 1 [file Data_Sheet_1.ZIP › Supplementary file/miRDeep2 file.zip!/miRDeep2 file/Know_Structure/SPLP42A3/oar-mir-150.pdf]

miRBase precursor : oar-mir-150
Total read count : 581
oar-miR-150 read count : 579
remaining reads : 2

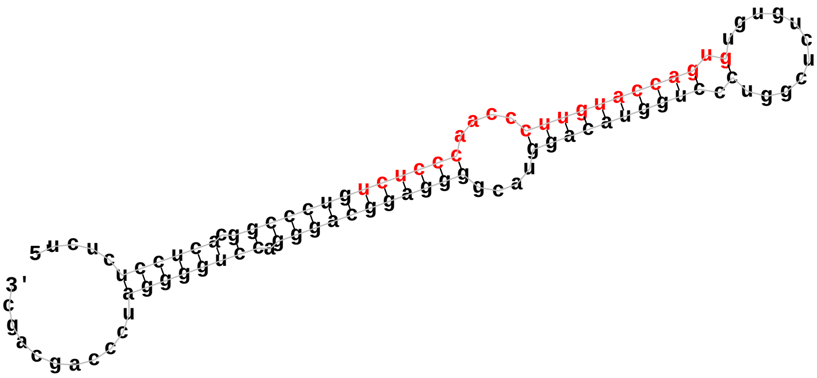

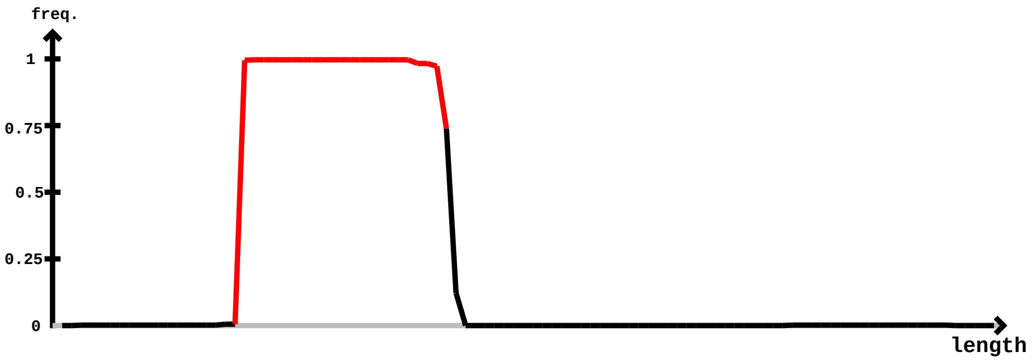

oar-miR-150

```
5'- ucucuccucacggcccugucucccaacccuuguaccagugugugucucggucccugguacagguacggggaggcagggaccuggggaucccagcagc   -3'    exp
    ....((((((.(((((((((((((....((((((((((.(...........).))))))))))....))))))))))).))))))))..........    reads      mm         sample
    .cucuccucacggcccuU...............................................................................    1          1          seq
    ................ugucucccaacccuuguaccagu..........................................................    2          0          seq
    ..................ucucccaacccuuguacc.............................................................    8          0          seq
    ..................ucucccaacccuuguaccag...........................................................    6          0          seq
    ..................ucucccaacccuuguaccagu..........................................................    133        0          seq
    ..................ucucccaacccuuguaccagug.........................................................    358        0          seq
    ..................ucucccaacccuuguaccagugu........................................................    71         0          seq
    ...................cucccaacccuuguaccagu..........................................................    1          0          seq
    ...........................................................................gggaccuggggauccca.....    1          0          seq
```
